# Blockade of the *N*-Methyl-D-Aspartate Glutamate Receptor Ameliorates Lipopolysaccharide-Induced Renal Insufficiency

**DOI:** 10.1371/journal.pone.0132204

**Published:** 2015-07-02

**Authors:** Chian-Shiung Lin, Shun-Fa Hung, Ho-Shiang Huang, Ming-Chieh Ma

**Affiliations:** 1 Department of Surgery, Liou-Ying Hospital, Chi-Mei Medical Center, Tainan Hsien, Taiwan; 2 Department of Urology, Far Eastern Memorial Hospital, New Taipei, Taiwan; 3 Department of Urology, National Cheng Kung University Hospital, Tainan, Taiwan; 4 School of Medicine, Fu Jen Catholic University, New Taipei, Taiwan; University of São Paulo School of Medicine, BRAZIL

## Abstract

N-methyl-D-aspartate (NMDA) receptor activation in rat kidney reduces renal perfusion and ultrafiltration. Hypoperfusion-induced ischemia is the most frequent cause of functional insufficiency in the endotoxemic kidney. Here, we used non-hypotensive rat model of lipopolysaccharide-induced endotoxemia to examine whether NMDA receptor hyperfunction contributes to acute kidney injury. Lipopolysaccharide-induced renal damage via increased enzymuria and hemodynamic impairments were ameliorated by co-treatment with the NMDA receptor blocker, MK-801. The NMDA receptor NR1 subunit in the rat kidney mainly co-localized with serine racemase, an enzyme responsible for synthesizing the NMDA receptor co-agonist, D-serine. The NMDA receptor hyperfunction in lipopolysaccharide-treated kidneys was demonstrated by NR1 and serine racemase upregulation, particularly in renal tubules, and by increased D-serine levels. Lipopolysaccharide also induced cell damage in cultured tubular cell lines and primary rat proximal tubular cells. This damage was mitigated by MK-801 and by small interfering RNA targeting NR1. Lipopolysaccharide increased cytokine release in tubular cell lines via toll-like receptor 4. The release of interleukin-1β from these cells are the most abundant. An interleukin-1 receptor antagonist not only attenuated cell death but also abolished lipopolysaccharide-induced NR1 and serine racemase upregulation and increases in D-serine secretion, suggesting that interleukin-1β-mediated NMDA receptor hyperfunction participates in lipopolysaccharide-induced tubular damage. The results of this study indicate NMDA receptor hyperfunction via cytokine effect participates in lipopolysaccharide-induced renal insufficiency. Blockade of NMDA receptors may represent a promising therapeutic strategy for the treatment of sepsis-associated renal failure.

## Introduction

The N-methyl-D-aspartate (NMDA) receptor is an ionotropic receptor/calcium channel within the CNS that is activated by the excitatory neurotransmitter, glutamate, to perform critical functions that control synaptic plasticity during learning and memory formation [1]. The NMDA receptor is also expressed in extraneural tissues including the kidney [2–8], where its functions are less well-known. Enhanced NMDA receptor function induced by channel overexpression mediates cytotoxicity due to massive calcium influx [1]. The entry of calcium through NMDA receptors is mainly gated by the NR1 subunit, which forms a tetramer with other modulatory subunits [1]. Different NMDA receptor subunits are present in the glomeruli, arterioles, and tubules of the rat kidney [4–8]. In addition, the glutamate recognition site on the NR1 subunit, D-serine, is thought to bind stereo-selectively to the glycine-regulatory site. The effects on NMDA receptor activation in motor neurons are either equal to or 100-fold more potent than those of glycine [9]; thus D-serine may be a physiological co-agonist for receptor activation [10]. Furthermore, D-serine is endogenously synthesized from L-serine by the enzyme, serine racemase (S-Race) [10]. We previously showed that S-Race is also present in the rat kidney [8], clearly indicating the presence of a complete NMDA receptor system.

The effect of NMDA receptors on renal hemodynamic regulation, however, is unclear. Inhibition of NMDA receptors by systemic application of MK-801 (a channel blocker) and 5,7-dichlorokynurenic acid (a glycine antagonist) induces renal vasoconstriction and attenuates renal vasodilatory responses to glycine infusion, indicating that renal NMDA receptors act as vasodilators [5]. We previously showed that direct activation of renal NMDA receptors by intrarenal arterial infusion of NMDA decreases the glomerular filtration rate (GFR) and urine and salt excretion [7], indicating that renal NMDA receptors act as vasoconstrictors. Different intensities and durations of NMDA receptor activation may explain the discrepancy between these observations, suggesting that renal NMDA receptors may play a role in hemodynamic regulation. Interestingly, renal NMDA receptor hyperactivity contributes to kidney injury caused by channel overexpression, as demonstrated in disease models utilizing short-term treatment with the nephrotoxic drug gentamicin or ischemia-reperfusion [7, 11].

Despite recent advances in medical treatment, the overall mortality of sepsis caused by multiple organ failure remains high [12–14]. Patients with sepsis often suffer acute renal failure [14], so identifying molecular targets that will enable effective treatment of sepsis-related kidney dysfunction is therefore of utmost importance. NMDA receptor inhibition attenuates hippocampal neuronal degeneration and reduces inflammation or oxidative stress in intestine, liver, and lung tissues of rat models of lipopolysaccharide (LPS)-induced endotoxemia or sepsis [15–17]. This suggests that NMDA receptor hyperfunction is involved in LPS-induced multiple organ failure. However, it is not known whether NMDA receptors influence LPS-induced renal insufficiency, although we previously showed that LPS impairs renal function via increased inflammatory cytokine release [18].

The aim of the present study was to examine whether NMDA receptor hyperfunction exacerbates renal excretory function in a rat model of LPS-induced endotoxemia. Since NR1 is expressed in porcine kidney epithelial cells (LLC-PK_1_) and Madin-Darby canine kidney (MDCK) cells [4], which represent cells of proximal and distal tubule origin, respectively. The direct effect of LPS on these cells and on proximal tubular cells isolated from rat kidneys was also tested as a model of tubular cell damage.

## Materials and Methods

### Experimental animals

Experiments were performed using male Wistar rats (BioLasco, Taipei, Taiwan) weighing approximately 200–250 g. All animal studies were reviewed and approved by the Institutional Animal Care and Use Committee of Fu Jen Catholic University (permit numbers: A10128 and A10247), and animal care and experimental protocols were performed in accordance with the guidelines for Care and Use of Laboratory Animals (National Academy Press, Washington DC, 2011). All surgery was performed under sodium pentobarbital anesthesia, and all efforts were made to minimize suffering.

### Induction of endotoxemia and NMDA receptor inhibition

Rats received an intraperitoneal injection of LPS (4 mg kg^-1^; Escherichia coli serotype 055:B5; Sigma-Aldrich, St. Louis, MO) to induce endotoxemia, as previously described [18]. These rats were termed the “LPS group”. Control groups received identical volumes of phosphate-buffered saline (PBS, pH 7.4). Basic body data and hemodynamic parameters were evaluated at 8, 24, and 48 h post-LPS injection. To examine the role of NMDA receptors, MK-801 (Sigma-Aldrich) was co-administered to LPS-treated (the LPS+MK-801 group) and PBS-treated (the MK-801 group) rats (0.3 mg kg^-1^ via subcutaneous injection), as previously described [19]. This dose did not produce obvious side effects, such as behavioral changes [19], and the final concentration in treated animals was comparable with that reported in a previous study [20].

### Surgical preparation for hemodynamic measurements

After induction, rats were anesthetized with sodium pentobarbital (50 mg kg^-1^, i.p.) and underwent surgical preparation for measurement of systemic and renal hemodynamics and urine collection as previously described [7, 21]. The PE-50 catheter was placed into the left carotid artery for blood sampling and for continuous measurement of the systemic mean arterial blood pressure (MABP). The heart rate (HR) was measured by arterial pulse wave analysis using MP36 AcqKnowledge software (Biopac, Los Angeles, CA). Another catheter was inserted into the left femoral vein to facilitate saline supplementation. The left kidney was exposed via a flank incision and the ureter was cannulated by PE-10 catheter for urine collection. The renal blood flow (RBF) was measured using an ultrasound flow probe (Transonic Systems, Ithaca, NY). Regional blood perfusion in the renal cortex of the left kidney, termed cortical microvascular blood flow (CMBF), was monitored using a flowmeter (Transonic Systems). All hemodynamic parameters were continuously recorded and displayed on a monitor. Arterial blood (200 μl) was then sampled for creatinine assays.

### Measurement of renal function

Following a 1 h recovery period, the rats were prepared for renal clearance studies [7]. Saline, containing 2% inulin (BioPAL, Inc., Worcester, MA), was administered intravenously (1.2 ml h^-1^) throughout the experiment. An arterial blood sample (200 μl) was obtained from the carotid arterial catheter half way through a 1 h clearance period.

### Biochemical assays and urinalysis

All assays were performed as described previously [7, 18, 21, 22]. Plasma creatinine levels were measured using a commercial kit (Bio-Quant, San Diego, CA). Urine volume was estimated gravimetrically to determine the urinary flow rate, and hematocrit was measured by a capillary tube after centrifugation. The inulin concentration in urine and plasma was measured by spectrophotometry, and the GFR was estimated according to inulin clearance. Sodium levels in urine or plasma were measured using flame photometry to determine the excretory rate of sodium and fractional excretion of sodium. Fractional excretion of sodium (%) was calculated as [urinary excretion rate of sodium/(plasma sodium concentration × GFR)] × 100. Renal filtration fraction (%) was calculated as {GFR/[RBF×(1- hematocrit)] ×100}. Urinary levels of protein, α-GST, and μ-GST were measured using commercial kits, as previously described [23].

### Tubular cell cultures and drug treatment

For the *in vitro* assays, LLC-PK_1_ and MDCK cells were used as a model of proximal and distal tubule cells, respectively, and rat proximal tubular cells were isolated for use as the primary culture model. LLC-PK_1_ and MDCK cells were purchased from the Bioresource Collection and Research Center (Hsinchu, Taiwan). Tubular cell lines were originally derived from the American Type Culture Collection lines CL-101 (for LLC-PK_1_) and CCL-34 (for MDCK). All culture medium and supplements were purchased from Thermo Scientific HyClone (South Logan, UT). LLC-PK_1_ cells were cultured at 37°C/5% CO_2_ in Medium 199 containing 3% fetal bovine serum, sodium bicarbonate (1.5 g l^-1^), penicillin (10,000 U ml^-1^) and streptomycin (10,000 μg ml^-1^). Cells were subcultured when they reached confluence (approximately every 2–3 days). MDCK cells were cultured in Eagle’s Minimum Essential Medium containing 10% fetal bovine serum, 2 mM L-glutamine, 1.5 g l^-1^ sodium bicarbonate, 0.1 mM nonessential amino acids, 1 mM sodium pyruvate, and the same concentrations of penicillin and streptomycin. Cells were treated with PBS, LPS (50 μg ml^-1^), MK-801 (1 μM), a TLR-4 antagonist (LPS from *Rhodobacter sphaeroides*, LPS-RS; 20 ng ml^-1^; Sigma-Aldrich), an IL-1 receptor antagonist (IL-1Ra, 10 ng ml^-1^; Sigma-Aldrich), or a combination of these for 4, 8, or 24 h. Dosing was determined by reference to the IC_50_ of each antagonist.

Proximal tubular cells were isolated from rat kidneys by enzymatic digestion using a commercial kit (Chi Scientific, Maynard, MA). Kidneys were removed from anesthetized rats and washed with ice-cold tissue washing medium (modified DMEM medium containing amphotericin, gentamycin, and fetal bovine serum) to remove blood. Renal cortical tissue was collected with the help of a stereomicroscope (Olympus, Center Valley, PA). Cortical samples were sliced into pieces (~1 mm wide) and digested with tissue dissociation medium containing collagenase and trypsin at 37°C for 30 min with stirring. The suspension was then filtered through a sieve with a mesh size of 80 μm. The cells were collected from the sieve after centrifugation and then suspended in basal culture medium containing fibroblast growth inhibitor, rat epidermal growth factor, hydrocortisone, and penicillin/streptomycin. Cells were plated at an appropriate cell density in a collagen-coated dish and then cultured at 37°C/5% CO_2_. The culture medium was changed after 24 h to remove nonadherent cells and residual cellular fragments. After 5 days, the cell cultures with 80% of confluent were treated with LPS or siRNA.

### RNA interference for NR1

A commercially available siRNA targeting NR1 (Ambion, Life Technologies, Carlsbad, CA) was used according to the manufacturer's instructions. Tubular cells were plated in 96-well plates. When the cells reached 80% confluence, the culture medium was replaced with fresh serum- and antibiotic-free medium. The NR1 siRNA (Ambion) sense strand sequence was 5′-GGAGAAUAUCACUGACCCAtt-3′. Briefly, transfection reagent (siPROT, Ambion) and siRNA (final concentration, 30 nM) were mixed in serum-free medium and incubated at room temperature for 20 min. The mixture was then added to the cells in each well for 12 h of incubation before LPS treatment. After transfection, the culture medium was replaced with fresh medium prior to LPS treatment.

### Cell viability and assays

Commercial kits were used to measure the levels of lactate dehydrogenase (LDH) (Roche Applied Science, IN), IFN-γ, IL-1β, and TNF-α (R&D Systems, Inc., Minneapolis, MN) in the cell culture medium.

### Measurement of D-serine levels

An enzyme-linked immunosorbent assay was used to measure D-serine levels, as previously described [24, 25]. Renal tissues, brain cortex, and cell pellets were homogenized in ice-cold distilled water, and the supernatant of homogenized samples and culture medium were collected for use in the assay. The protein concentration in sample was determined using a colorimetric assay kit from Bio-Rad (Hercules, CA). Glutaraldehyde (25 μl) was added to 100 μl of each extract (containing 1 mg ml^-1^ sample protein) or to 100 μl of a D-serine standard (0.01–10 nM) in PBS containing 0.2 mg ml^-1^ BSA. The D-serine standard solutions and samples containing glutaraldehyde were mixed thoroughly, and 50 μl of this solution was added to a 96-well plate in duplicate. After 2 h of incubation at room temperature, the wells were washed with PBS containing Tween 20 (PBST) and air dried. Blocking buffer (2% nonfat milk in PBST; 50 μl) was added to each well and incubated for 30 min at room temperature. The plates were then washed three times in PBST (300 μl per well). A D-serine-specific antibody (Santa Cruz Biotechnology, Dallas, TX) was diluted 1:1,000 in PBST containing 1% BSA. Fifty microliters of this solution was then added to each well and incubated for 1 h, followed by three washes with PBST. A horseradish peroxidase-linked anti-rabbit antibody (Santa Cruz Biotechnology) was then added for 30 min, followed by three washes in PBST. A solution of 2,2'-azinobis [3-ethylbenzothiazoline-6-sulfonic acid]-diammonium salt (50 μl, Sigma-Aldrich) was added to each well and the resulting colorimetric reaction was measured at 405 nm. The amount of D-serine in sample was expressed as nmole per mg of protein.

### Detection of protein and mRNA expression

Western blotting was performed using antibodies (Santa Cruz Biotechnology) against NR1 (1:1000), S-Race (1:5000), TLR4 (1:2000), or actin (1:5000), as described previously [8].

Semi-quantitative or real-time RT-PCR was used to detect NR1 or S-Race mRNA expression, as previously described [8]. Complementary DNA (cDNA) was synthesized using 10 μg of total RNA and amplified, as previously described (8). The NR1 primer sequences for conventional PCR were as follows: 5’-ACG GAA TGA TGG GCG AGC-3’ (sense) and 5’-GGC ATC CTT GTG TCG CTT GTA G-3’ (antisense) (transcript product, 1,033 bp; NM_017010). The S-Race primers were as follows: 5’-CCC AAA GCC GTT GTT ACT CAC A-3’ (sense) and 5’-CAT TGG AAG GTT CAG CAG CGT ACA-3’ (antisense) (395 bp; NM_198757). Actin primers were as follows: 5’-TCA GGT CAT CAC TAT CGG G-3’ (sense) and 5’-CAG TAA TCT CCT TCT GCA TC-3’ (antisense) (221 bp; NM_031144.3). PCR began with a denaturation step of 1 min at 94°C followed by 40 cycles for NR1, S-Race, and actin at 94°C for 30 s, 58°C for 1 min, and 72°C for 1 min. The PCR products were electrophoresed on a 2% agarose gel and visualized by ethidium bromide staining. The densities of the bands were measured using an image analytic system (Diagnostic Instruments), and mRNA levels are expressed as the ratio of NR1 or S-Race to actin. The primer sequences for real-time RT-PCR were listed in S1 Table. The comparative C_T_ (ΔΔC_T_) method was used to quantify NR1 mRNA levels. The calculation used was: ΔΔC_T_ = [C_T_ NR1 (unknown sample)—C_T_ GAPDH (unknown sample)]—[C_T_ NR1 (calibrator sample)—C_T_ GAPDH (calibrator sample)]. The cDNA of calibrator sample was obtained from rat brain cortex.

Indirect immunofluorescent staining was used to examine the cellular distribution of FLK-1 (Santa Cruz Biotechnology), NR1, or S-Race and their co-localization in renal tissues or tubular cells, as described previously [8]. Briefly, postfixed kidneys were stored in 10% sucrose prepared in 4% paraformaldehyde solution at 4°C, embedded in O.C.T. compound (Tissue-Tek, Sakura Finetek, Torrence, CA), and frozen at –20°C, and then cut into 5 μm sections on a cryostat (Microm, Heidelberg, Germany), which were thaw-mounted on coated slides. Cells were cultured on poly-D-lysine-precoated glass dishes and postfixed in 4% paraformaldehyde solution at 4°C for 2 h. After being rehydrated and washed with PBS, tissue sections and culture dishes were processed for indirect immunofluorescence using a tyramide signal amplification kit (PerkinElmer, Waltham, MA). After being blocked with 5% skimmed milk prepared in PBS for 1 h, sections were incubated overnight at 4°C with an anti-NR1 antibody and then for 1 h at room temperature (RT) with the corresponding cyanine 3-conjugated secondary antibody. After detection of NR1, tissue sections or culture dishes were incubated overnight at 4°C with an anti-S-Race or anti-FLK-1 antibody, and then for 1 h at RT with the corresponding fluorescein-conjugated secondary antibody, and examined on an inverted microscope (Leica Microsystems GmbH, Wetzlar, Germany) equipped with a fluorescence image analytic system (Diagnostic Instruments, Sterling Heights, MI). Nuclei were counterstained using DAPI. The specificity of each antibody was tested by preincubation with the specific blocking peptide provided by Santa Cruz Biotechnology (150 μg ml^-1^) before performing the test.

### Statistical analysis

Data are expressed as the mean ± standard error of the mean (SEM). Plasma creatinine, proteinuria, and enzymuria data that are not normally distributed are expressed as median and interquartile range (IQR). Statistical analysis was performed using analysis of variance coupled with Bonferroni post hoc analysis or non-parametric Mann-Whitney test for multiple comparisons between groups. A P value of < 0.05 was considered significant.

## Results

### NMDA receptor inhibition attenuates LPS-induced impairment of renal function

**Fig 1:**
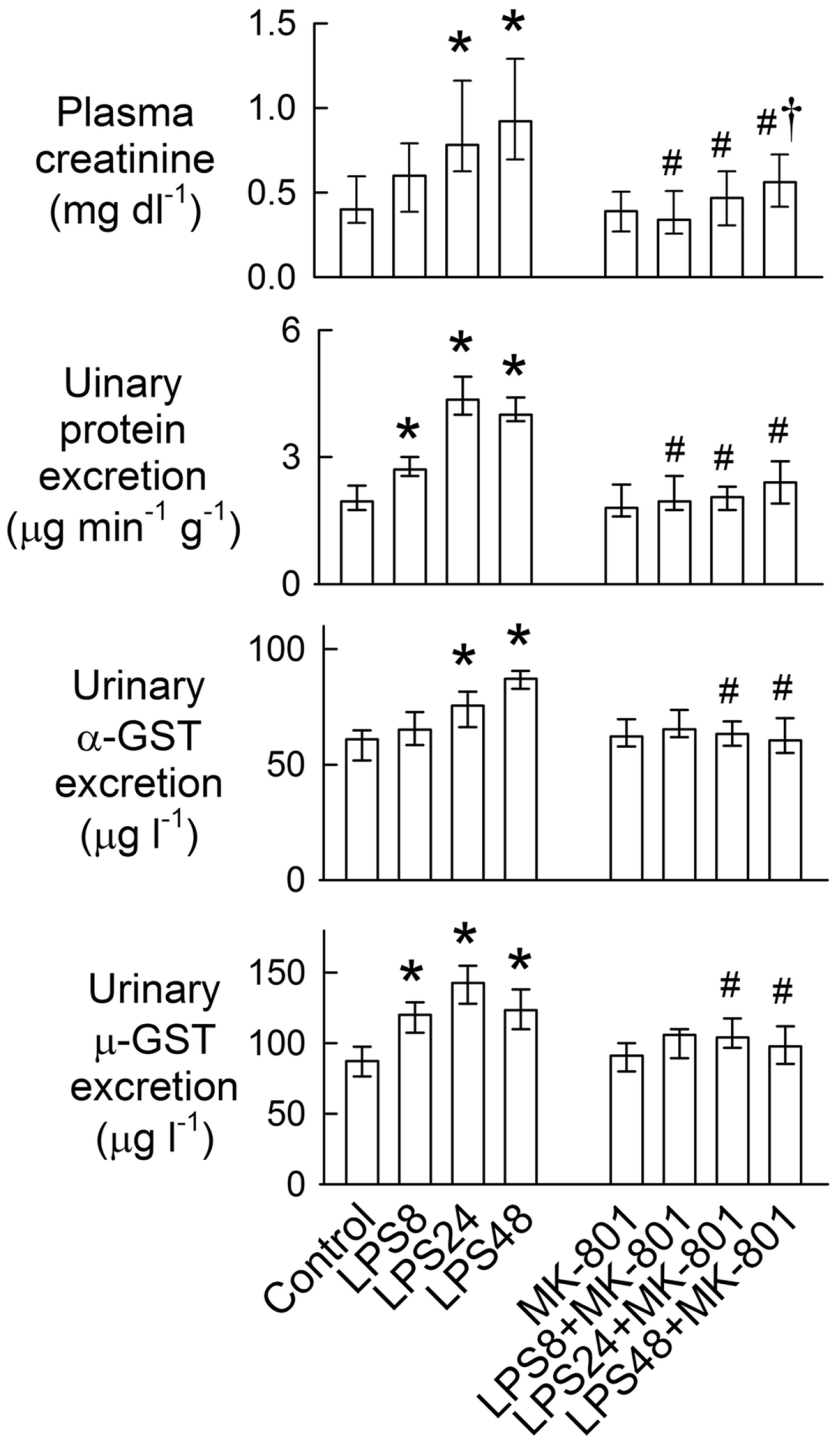
Effects of MK-801 on lipopolysaccharide (LPS)-mediated changes in plasma creatinine, proteinuria, and enzymuria. The bar represents 50% of the values (median) with the upper bar representing the 75th percentile (Q_3_) and the lower bar representing the 25th percentile (Q_1_). N = 8 in each group. *P < 0.05 *vs*. the control groups. ^#^P < 0.05 *vs*. the LPS group at the same time-point. †P < 0.05 *vs*. the MK-801 group.

Compared with the control group, LPS increased plasma creatinine levels at 24 and 48 h (Fig 1) and this was associated with increased urinary protein excretion at all time-points (P < 0.05). Moreover, LPS significantly increased urinary excretion of the specific tubular markers, α-glutathione S-transferase (α-GST, a proximal tubular cell marker of cytoplasmic leakage) and μ-GST (a distal tubular cell marker), at 4–48 h and at 24–48 h, respectively. Body weight, left kidney weight, hematocrit, and plasma sodium concentration were similar for all groups (S2 Table). MK-801 reduced LPS-induced increases in plasma creatinine, with a significant effect observed at 48 h. MK-801 also abrogated LPS-induced enzymuria.

**Fig 2:**
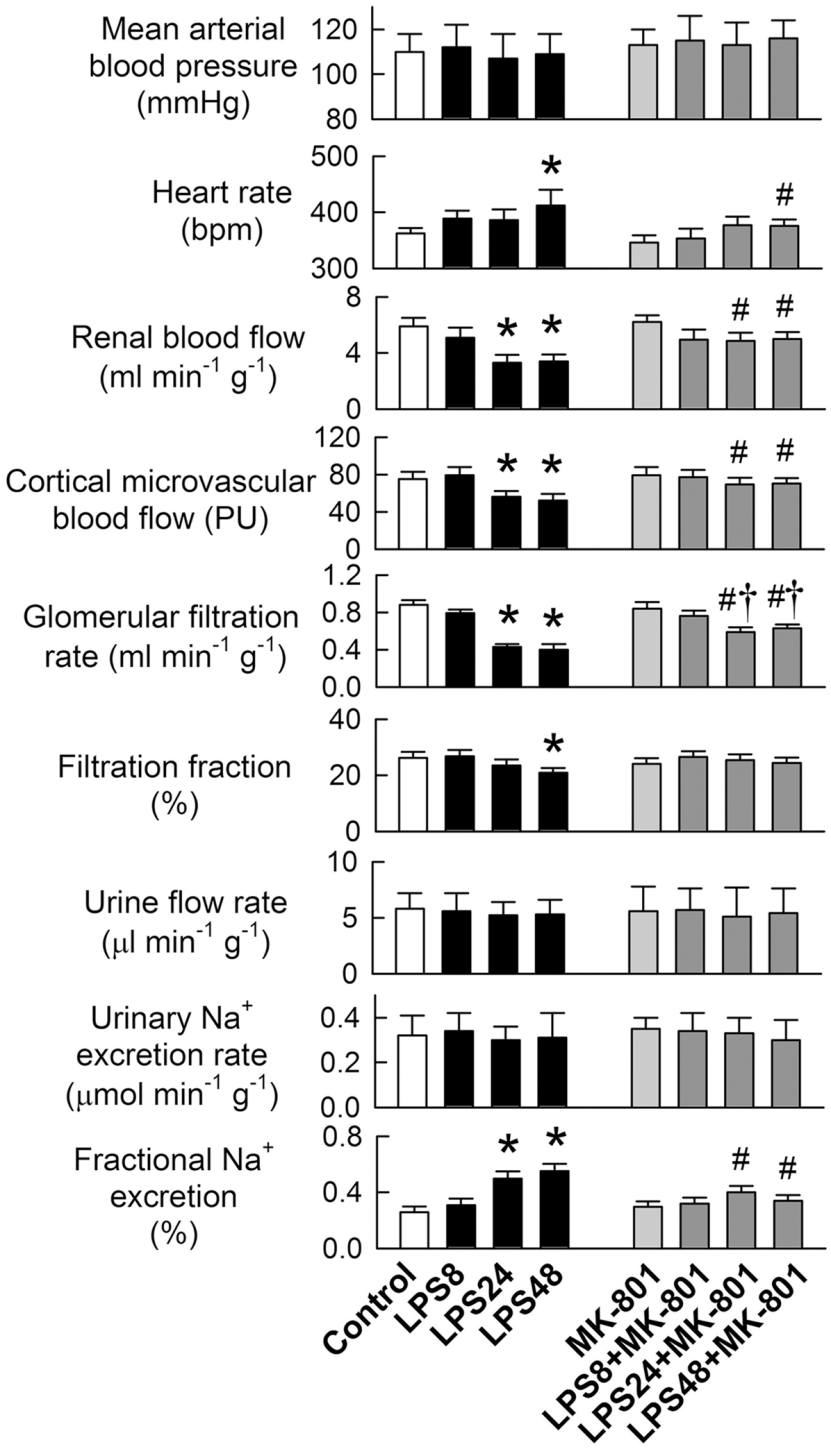
Effects of MK-801 on lipopolysaccharide (LPS)-mediated renal functional impairment. N = 8 in each group. *P < 0.05 *vs*. the control groups. ^#^P < 0.05 *vs*. the LPS group at the same time-point. †P < 0.05 *vs*. the MK-801 group.

There were no differences in MABP among groups (Fig 2). MK-801 abolished tachycardia in the LPS48 group. LPS impaired renal function in the left kidney by reducing RBF, CMBF, GFR, and filtration fraction at 24 or 48 h, but not at 8 h. MK-801 significantly improved the LPS-induced functional decline, although low GFRs were still seen at 24 and 48 h. Baseline urinary output and Na^+^ excretion were similar among groups. MK-801 reversed the LPS-induced increases in fractional excretion of sodium at 24 and 48 h, indicating NMDA receptor inhibition ameliorates sodium-wasting.

### Detection of NR1 and S-Race expression in renal tissues

**Fig 3:**
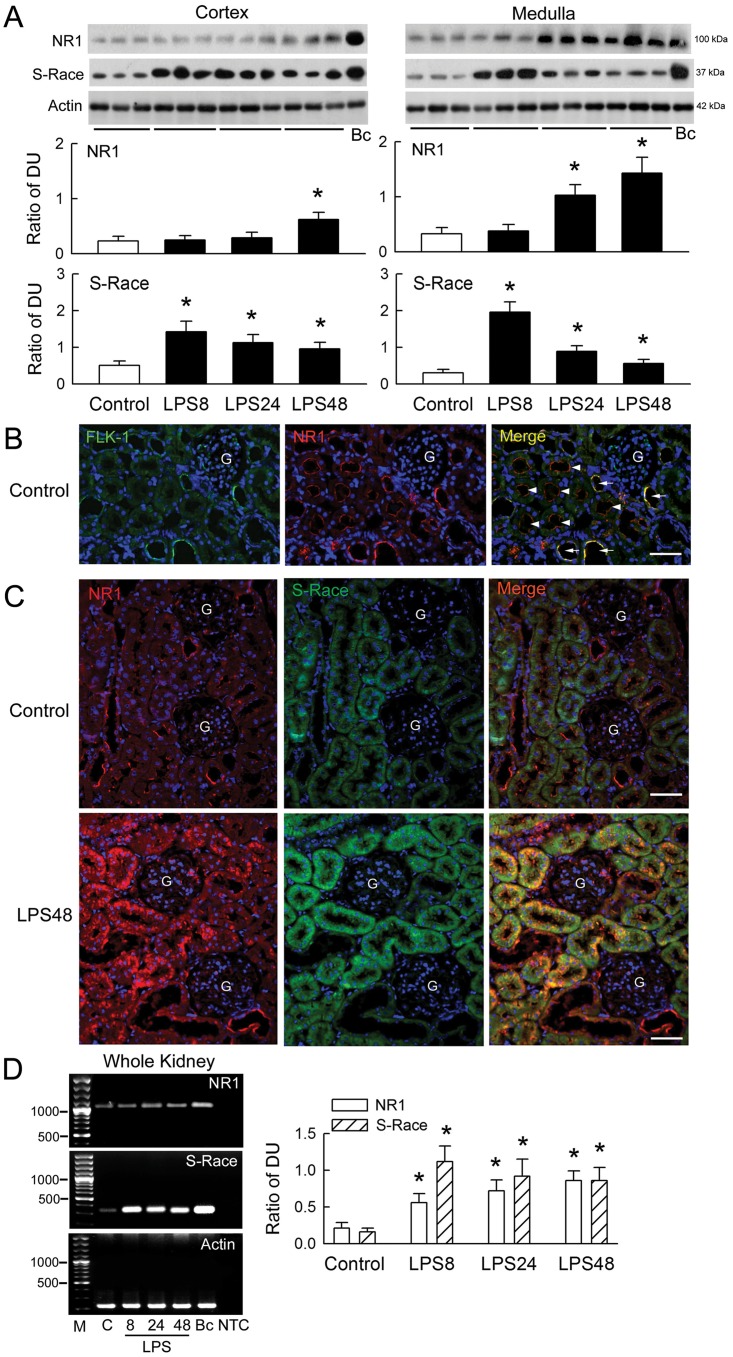
Changes in NR1 and S-Race expression in the rat kidney. (A) The upper panels show representative blots from control and lipopolysaccharide (LPS)-treated kidneys at 8, 24, and 48 h (n = 3), together with a positive sample obtained from brain cortex (Bc). Bar graphs show the ratio of NR1 or S-Race to actin [expressed as densitometric units (DU)] (n = 8). (B) Representative micrographs of FLK-1- (green) and NR1- (red) positive immunoreactivity, and co-localized immunofluorescence (yellow color in merged picture) in the control kidney. Nuclei were counterstained with DAPI (blue). G, glomerulus. The horizontal bars in merged pictures indicate 150 μm. (C) Representative pictures of NR1- (red) and S-Race- (green) expression, and co-localization (orange color in merged picture) in the control kidney (upper) and LPS48 kidney (lower). Magnification: ×200 in B and C. (D) Right panels: representative gels showing NR1 and S-Race mRNA expression. M, 100 bp DNA ladder; C, control; Bc, brain cortex; NTC, no template control. The bar graphs on the left show the DU ratio (n = 8). *P < 0.05 *vs*. the control group.

LPS caused a gradual and time-dependent increase in NR1 expression in the renal cortex and medulla (Fig 3A). By contrast, LPS caused an abrupt increase in S-Race expression after 8 h of treatment, which remained high thereafter. Immunostaining identified NR1 in the endothelial lining of renal vessels (marked by FLK-1, a vascular endothelial growth factor receptor, Fig 3B) and at the apical membrane of the proximal tubules and collecting ducts, with a more limited distribution in the glomeruli of control kidneys (Fig 3C). S-Race was mostly localized in the renal tubules, although there was some expression in the renal vessels. No signal was observed in the glomeruli. NR1 mainly co-localized with S-Race. Compared with that in the control group, there was a marked increase in the NR1 signal in the tubules and vessels in LPS48-treated kidneys; however, increased S-Race expression was only observed in tubules in which NR1 was also present. LPS treatment also increased the expression of NR1 and S-Race mRNA (Fig 3D).

### Effects of LPS on renal tubular cells

**Fig 4:**
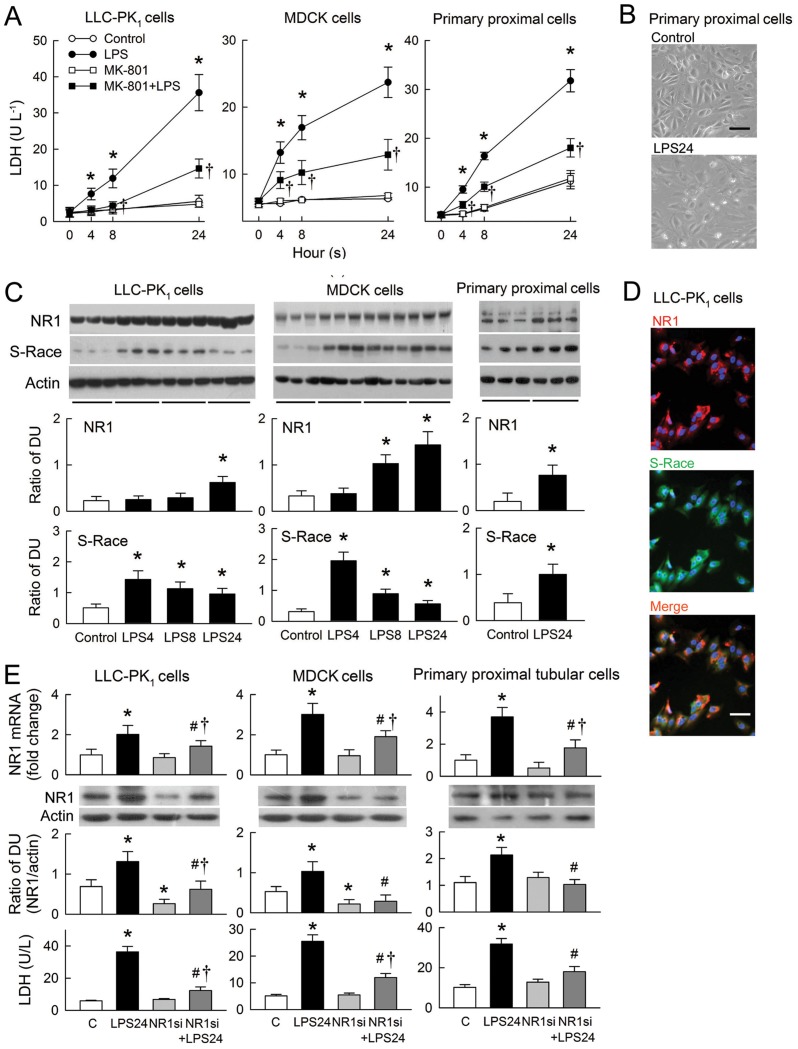
Effects of lipopolysaccharide (LPS) on renal tubular cells. (A) Panels show lactate dehydrogenase (LDH) release in LLC-PK_1_ (left), MDCK (middle), and primary rat proximal tubular cells (right) treated with LPS, MK-801, or PBS (control) (n = 6). (B) Representative images showing morphological changes in primary rat proximal tubular cells treated with either PBS (control) or LPS for 24 h. (C) Representative blots of NR1, S-Race, and actin obtained from three separate culture wells. The bar graphs below show the densitometry data from six assays. (D) Representative images of immunofluorescence staining for NR1 (red) and S-Race (green) in untreated LLC-PK_1_ cells. Co-localized staining is orange (merged picture). Nuclei were counterstained with DAPI (blue). Magnification: ×400 in B and D. The horizontal bar in merged picture indicates 10 μm. (E) The upper bar graphs show silencing of NR1 mRNA as analyzed by real time RT-PCR. Results were expressed as fold changes with respect to LLC-PK_1_ (left column), MDCK (middle column), and primary rat proximal tubular cells (right column) receiving a negative control siRNA (C) (n = 6). The middle representative blots and bar graphs (n = 6) show changes in protein expression of NR1 in tubular cells after LPS treatment with or without NR1 siRNA by Western blot analysis. The lower bar graphs show the effects of NR1 siRNA on LDH release in tubular cells treated with various combinations of LPS or siRNA (n = 6). *P < 0.05 *vs*. the control (C) groups. ^#^P < 0.05 *vs*. the LPS group at the same time-point or the LPS24 plus NR1 siRNA (NR1si)-treated group. †P < 0.05 *vs*. the MK-801 group or the NR1 siRNA-treated group.

LPS-induced cytotoxicity in tubular cells was examined in a LDH release assay (Fig 4A). LPS induced a gradual increase in LDH release from both LLC-PK_1_ and MDCK cells, and from a primary culture of rat proximal tubular cells. LPS-induced LDH release was markedly attenuated by MK-801. MK-801 alone had no effect on LDH release. In the absence of LPS, rat proximal tubular cells showed the typical cobblestone appearance of a normal epithelial cell monolayer (Fig 4B). When the cells were exposed to LPS for 24 h, a number of cells became swollen and enlarged, a typical feature of necrosis. LPS increased the expression of both NR1 and S-Race in all tested cells (Fig 4C). The increase in NR1 expression in LLC-PK_1_ and MDCK cells was gradual (over 24 h), whereas S-Race expression peaked after 4 h of treatment and remained high thereafter. Immunocytostaining revealed the presence of NR1 and S-Race in untreated LLC-PK_1_ cells, with co-localization of most signals (Fig 4D). We next determined whether small interfering RNA (siRNA) targeting NR1 could silence the LPS-mediated upregulation of NR1. LPS significantly increased NR1 expression at mRNA and protein levels and LDH release in all tested cells after 24 h of treatment (Fig 4E). NR1 silencing alone showed no effect on NR1 mRNA expression but reduced NR1 protein expression in LLC-PK_1_ and MDCK cells. Both mRNA and protein expression of NR1 in cells treated with NR1 siRNA followed by LPS was reduced to levels lowered or similar to that in control cells, and these were associated with a reduction in LPS-induced LDH release.

### Changes in D-serine levels

**Fig 5:**
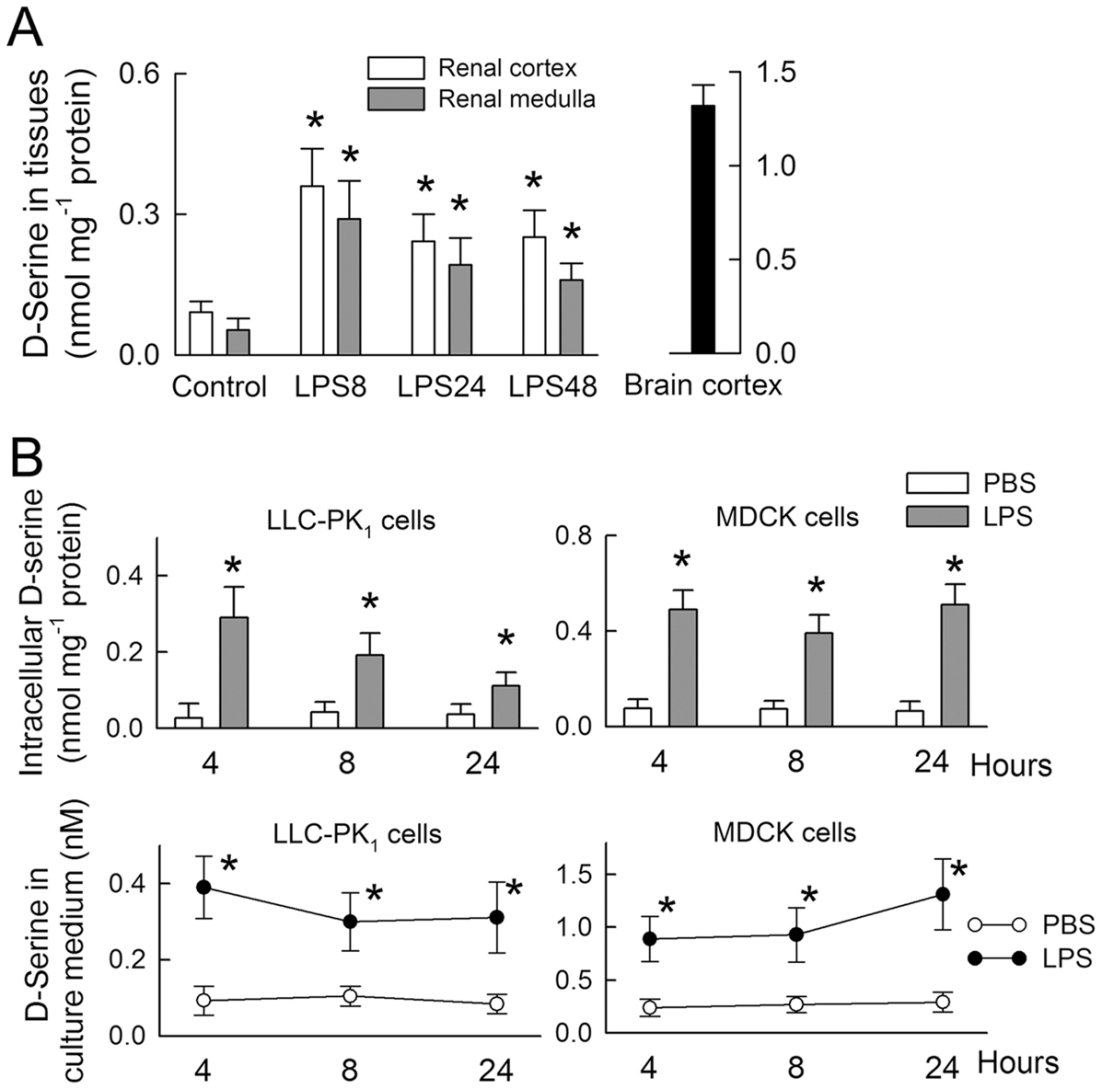
Changes in D-serine levels in renal tissues and tubular cells. (A) D-serine levels in rat kidneys following lipopolysaccharide (LPS) or control treatment (n = 8). Bc, brain cortex. (B) Intracellular D-serine levels (upper panels) and levels of D-serine released into the culture medium (lower panels) following LPS or control treatment (n = 5). *P < 0.05 *vs*. the control (PBS-treated) group at the same time-point.

To measure D-serine levels, a standard curve was generated using D-serine/BSA conjugates. This demonstrated a linear relationship between D-serine concentration (within a range of 0.01–10 nM) and optical density (S1 Fig). D-serine was detected in control kidneys at 10.5 ± 6.3% (renal cortex) and 6.8 ± 3.4% (renal medulla) of the levels detected in the cerebral cortex (Fig 5A). LPS treatment increased renal D-serine levels, with a maximal response observed after 8 h. D-serine was detected in cell lysates and culture medium from the tubular cell lines (Fig 5B). LPS treatment led to increases in intracellular D-serine and D-serine levels in the culture medium (all P<0.05), indicating its *de novo* synthesis and subsequent release.

### Cytokine effects in tubular cells

**Fig 6:**
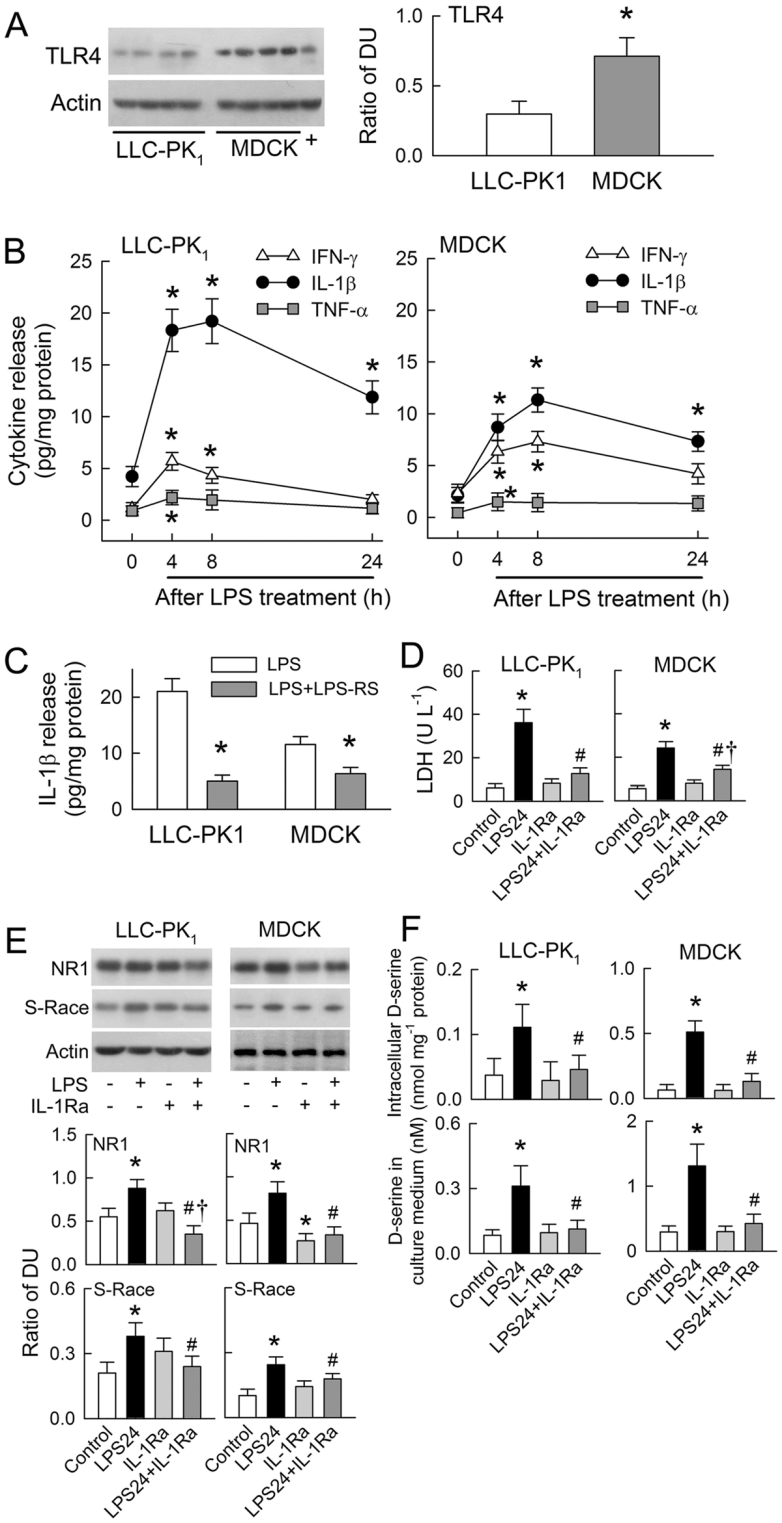
Cytokine effects of lipopolysaccharide (LPS)-induced N-methyl-D-aspartate (NMDA) receptor hyperfunction in tubular cells. (A) Representative blots of TLR4 expression (left) with densitometry data (right) pooled from four separate cultures. +, positive sample extracted from rat liver. (B) Graphs show IFN-γ, IL-1β, and TNF-α release into culture medium before (zero time-point) and after LPS treatment (n = 6) in both LLC-PK_1_ (left) and MDCK cells (right). (C) TLR4 antagonist LPS-RS were co-treated with LPS in tubular cells for 8 h. The release of IL-1β in medium was determined (n = 6). (D) IL-1 receptor antagonist (IL-1Ra) attenuates LPS-mediated LDH release after 24 h of treatment (n = 5). (E) Representative blots showing NR1, S-Race, and actin expression in cells treated with LPS, IL-1Ra, or a combination of the two. The lower panels show densitometry data pooled from five separate cultures. (F) Intracellular D-serine levels (upper panels) and D-serine levels in the culture medium (lower panels) at 24 h post-treatment with LPS, IL-1Ra, or a combination of the two (n = 5). *P < 0.05 *vs*. the LLC-PK_1_,the zero time-point, or the control groups. ^#^P < 0.05 *vs*. the LPS24 group. †P < 0.05 *vs*. the IL-1Ra group.

The LPS receptor, toll-like receptor 4 (TLR4), was detected in both cell lines with higher expression in MDCK cells than LLC-PK_1_ cells (Fig 6A). LPS increased the release of IFN-γ, IL-1β, and TNF-α in a time-dependent manner with peak expression around 4–8 h (Fig 6B). The increase in LPS-induced IL-1β release was the most prominent among the cytokines examined, especially in the LLC-PK_1_ cell line. Treatment with the TLR4 antagonist, LPS-RS, for 8 h markedly attenuated LPS-induced IL-1β release in LLC-PK_1_ and MDCK cells (Fig 6C). Co-treatment of both cell lines with LPS and IL-1 receptor antagonist (IL-1Ra) led to a significant reduction in LPS-induced cytotoxicity (by 79 ± 8% and 59 ± 6%, respectively) through suppressed LDH release (Fig 6D). In addition to reducing cytotoxicity, IL-1Ra also attenuated LPS-mediated increases in NR1 and S-Race expression in both cell lines (Fig 6E). Treatment of MDCK cells with IL-1Ra alone also reduced the expression of NR1. Interestingly, IL-1Ra abrogated the LPS-induced *de novo* synthesis and release of D-serine in both cell lines (Fig 6F).

## Discussion

**Fig 7:**
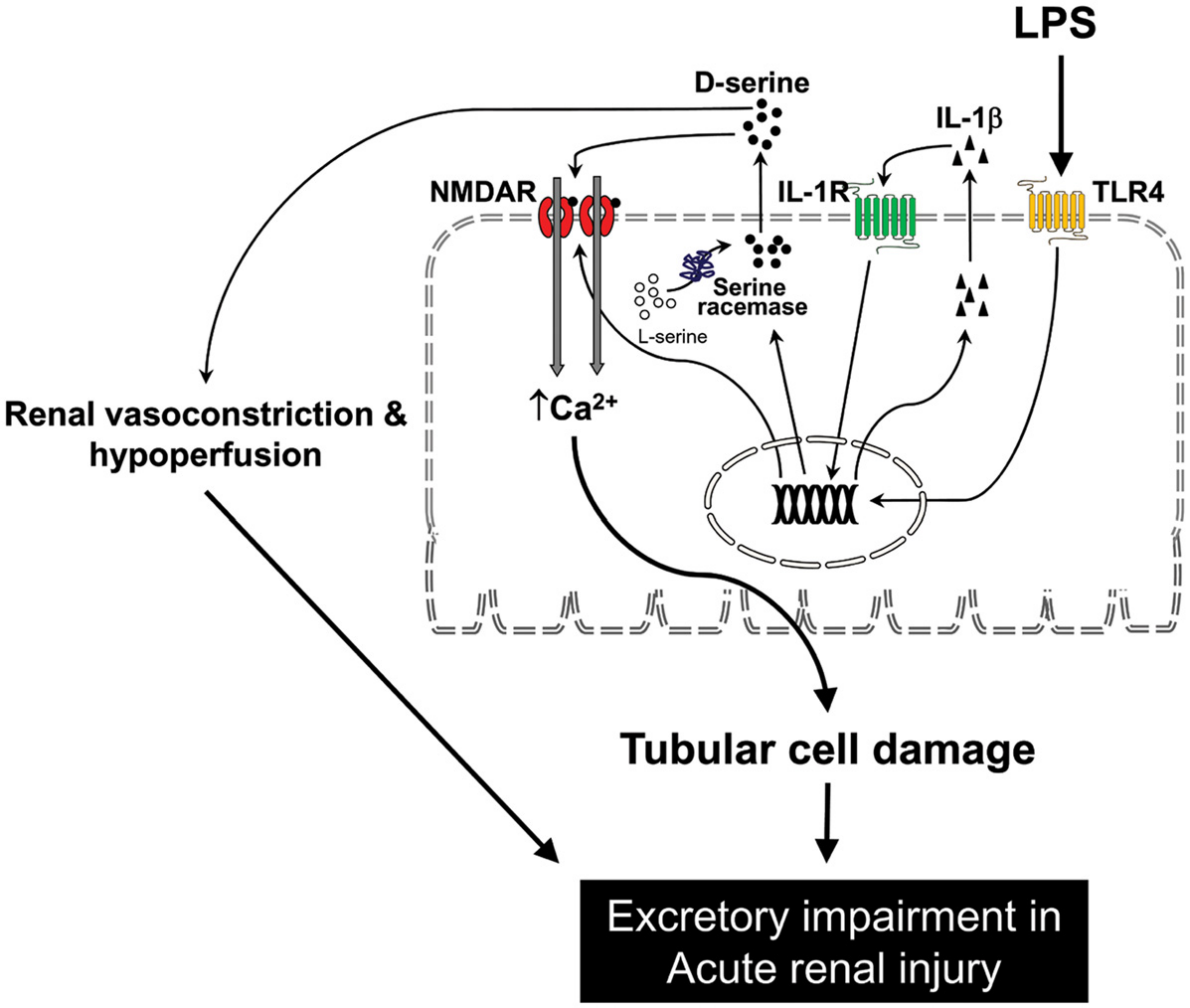
Schematic diagram showing how lipopolysaccharide (LPS) induces N-methyl-D-aspartate (NMDA) receptor hyperfunction and renal damage in endotoxemia. LPS binds to toll-like receptor 4 (TLR4) and induces interleukin (IL)-1β release from tubular cells. TLR4 and IL-1 receptor (IL-1R) signaling in tubular cells increases expression of the NMDA receptor NR1 subunit and serine racemase. Upregulation of NR1 and serine racemase, together with increased D-serine levels, results in NMDA receptor hyperfunction. NMDA receptor hyperfunction in LPS causes tubular cell damage and poor renal perfusion through vasoconstriction leading to acute renal failure.

As summarized in the schematic shown in Fig 7, this study provides novel evidence demonstrating that NMDA receptor hyperfunction contributes to acute renal failure during LPS-induced endotoxemia. LPS induced IL-1β release and caused upregulation of NR1 and S-Race, leading to NMDA receptor over-activation in tubular cells via S-Race-derived D-serine production and secretion. The poor renal perfusion and ultrafiltration in LPS-treated kidneys was due to the vasoconstrictor effects of D-serine on NMDA receptors. Inhibition of NMDA receptors attenuated LPS-induced functional deterioration and tubular cell damage in *in vivo* and *in vitro*.

Disturbances in systemic hemodynamic function during endotoxemia invariably lead to multiple organ failure due to inadequate tissue perfusion [18, 26]. The dose of LPS given in this study caused a significant reduction in renal perfusion but not in systemic arterial pressure (Fig 2). This is in agreement with previous results from a conscious rat model, which show that LPS decreases glomerular ultrafiltration without eliciting a reduction in blood pressure [26]. However, the apparent lack of any effect on blood pressure may be a compensatory result of tachycardia (Fig 2), which is caused by baroreceptor reflex-mediated sympathetic activation in response to LPS-mediated vasodilation [26]. Interestingly, NMDA receptors have been identified in (and are functionally present in) neural pathways involved in the reflex control of blood pressure and heart rate [27, 28]. We speculate that LPS may stimulate NMDA receptors within the CNS pathways that mediates tachycardia.

A previous *in vitro* study demonstrated that LPS selectively induces vasoconstriction in the renal artery, but not in the superior mesenteric artery, by impairing acetylcholine-induced endothelium-dependent relaxation in rats [29]. In this study, NMDA receptor inhibition prevented reductions in renal perfusion and ameliorated decline in ultrafiltration (Fig 2) but not NR1 expression (S2 Fig), indicating that NMDA receptor hyperfunction contributes to renal vasoconstriction in endotoxemic kidneys. This is consistent with our previous findings that direct activation of NMDA receptors (via intrarenal arterial administration of NMDA) reduced the GFR in a dose-dependent manner [18]; however, acute administration of NMDA had no effect on renal blood flow, which was decreased by LPS. The LPS-induced NMDA receptor upregulation in disease states may explain this difference. Nevertheless, the pathophysiological role of NMDA receptor hyperfunction in renal injury is well established. Expression of both renal NR1 protein and mRNA is upregulated during ischemia-reperfusion insult and by short-term treatment with gentamicin [7, 11], as well as in the endotoxemic model reported herein.

In addition to regulating renal vascular tone, the presence of NMDA receptors in renal tubules possibly might affect tubular reabsorption [4, 5, 30]. The immunostaining results presented herein support this view because NR1 was identified in both the proximal and distal tubules (Fig 3). We did not examine how LPS and NMDA receptors affect tubular transport. However, NMDA receptor blockade prevented sodium-wasting caused by LPS (Fig 2). Moreover, LPS enhances enzymuria of tubular markers *in vivo* and increases tubular cytotoxicity *in vitro* (Figs 1 and 4A), suggesting tubular cell damage or loss could be one of the mechanisms underlying salt-wasting after LPS treatment. The mechanism underlying LPS-mediated tubular damage is dependent on NMDA receptor hyperfunction because cell damage was ameliorated by MK-801 and NR1 silencing (Fig 4). Our hypothesis that LPS causes NMDA receptor hyperfunction is further supported by the observation that LPS upregulates NR1 and S-Race expression, and also by increases in D-serine levels in the rat kidney and in cultured tubular cells. Interestingly, the temporal changes in NR1 and S-Race expression observed in LPS-treated tubular cell cultures were similar to those observed in rat kidneys, indicating that most of the LPS-induced increases in protein expression were of tubular origin.

High plasma levels of D-serine are nephrotoxic because D-serine can be reabsorbed by straight segments of the proximal tubules, which causes tubular cell necrosis [31–34]. D-serine taken up by tubular cells is metabolized by an intracellular enzyme, D-amino acid oxidase, which generates reactive oxygen species via the depletion of reduced glutathione, thereby contributing to tubular damage [35]. It is not known whether there is an increase in plasma D-serine in LPS-treated animals; however, increased levels of D-serine in renal tissues (Fig 5) suggest a direct toxic effect on the kidney. This suggestion is supported by the *de novo* synthesis and release of D-serine by cultured tubular cells during LPS-mediated tubular cell damage (Fig 5). Since D-serine acts as an endogenous ligand for the NMDA receptor, and NR1 is present in renal tubules [1, 8], we speculate that D-serine-induced nephrotoxicity may be the result of NMDA receptor over-stimulation, as demonstrated by previous studies [31–35]. Whether the D-serine-mediated effects on NMDA receptor function influence nephrotoxicity warrants further study.

The LPS receptor, TLR4, is expressed in murine kidneys and shows a largely tubular distribution [36, 37]. TLR4 was also expressed in the tubular cell lines used herein, and its expression was higher in MDCK cells than in LLC-PK_1_ cells at the same amount of protein loading (Fig 6). It is therefore possible that MDCK cells may be more responsive to LPS than LLC-PK_1_ cells. However, LLC-PK_1_ cells released more LDH than MDCK cells in response to LPS (Fig 6), indicating that a mechanism downstream of TLR4 might be involved in LPS-induced tubular cell injury. Other studies have shown that TLR4 is the primary effector of LPS-mediated cellular responses, including the rapid release of cytokines [37]. This study shows that LPS enhances cytokine release in both of the tubular cell types studied (Fig 6). Among the cytokines released, IL-1β release was the most prominent (Fig 6). Moreover, TLR4 inhibition markedly attenuated IL-1β release with a 76% and 48% reduction in LLC-PK_1_ and MDCK cells, respectively. Though TLR4 was less abundant in LLC-PK_1_ than in MDCK cells, TLR4-mediated signaling (triggering IL-1β release) seemed to be more efficient in LLC-PK_1_ cells than in MDCK cells. Hence, LLC-PK_1_ cells appeared to be more sensitive to LPS treatment than MDCK cells. Interestingly, the results agree with previous findings that stimulation with toxic agents, such as oxalate, the environmental pollutant cadmium, and the nephrotoxic drug cyclosporine, results in stronger responses in LLC-PK_1_ cells than in MDCK cells [37–40].

IL-1 receptor inhibition completely abrogated LPS-induced cell injury, indicating that IL-1β is the main cytokine that triggers tubular cell death. We previously showed that a renoprotective strategy involving hypoxic preconditioning protects rat kidneys against the effects of LPS by attenuating IL-1β synthesis [18]. Interestingly, IL-1 receptor inhibition also attenuated LPS-induced upregulation of NR1 and S-Race, as well as D-serine synthesis and secretion (Fig 6), suggesting that IL-1β-mediated NMDA receptor hyperfunction could be the mechanism by which LPS induces tubular cell damage. This finding is corroborated by observations that signal pathways downstream of the IL-1 receptor and TLR4, including the activation of nuclear factor κB (NF-κB) and mitogen-activated protein kinases (MAPKs), induce LPS-mediated pleiotropic inflammatory responses [41]. NF-κB translocates to the nucleus during P19 cell differentiation into neurons and markedly increases NR1 promoter activity [42]. Another mechanism of relevance to NF-κB signaling is reactive oxygen species-mediated oxidative stress, which we previously demonstrated in LPS-treated kidneys [18]. Oxidative stress, induced by exposure to superoxide, peroxynitrite, and hydrogen peroxide, upregulates NR1 expression in bEnd3 cells, an endothelial cell derived from primary murine brain microvasculature [43]. This, however, can be completely blocked by an inhibitor of NF-κB activation [43]. It is therefore likely that LPS-mediated reactive oxygen species formation and NF-κB activation will have profound effects on NR1 upregulation. Although the S-Race gene is not a target for NF-κB, S-Race expression in MG5 microglial cells is upregulated by LPS through c-Jun N-terminal kinase MAPK signaling [44]. Further work is required to determine whether MAPKs via LPS affect S-Race expression in tubular cells.

In conclusion, the results presented herein show that LPS-induced renal injury is dependent on the NMDA receptor. Upregulation of NR1 and S-Race, together with increases in D-serine content, cause NMDA receptor hyperfunction in tubular epithelium after LPS treatment. This, in turn, induces renal vasoconstriction and hypoperfusion *in vivo* and further contributes to tubular cell death *in vitro*. Finally, LPS-dependent IL-1β release plays a critical role in NR1 and S-Race upregulation in tubular cells.

## Supporting Information

S1 Fig Standard curve generated using D-serine/bovine serum albumin conjugates and used to measure D-serine levels. The curve demonstrated a linear relationship between D-serine concentration (within a range of 0.01–10 nM) and optical density (O.D.) at 405 nm. Each data point is representative of five experiments.
(JPG)

S2 Fig Effects of NMDA receptor inhibition on NR1 expression in the rat kidneys. The upper panels show representative blots from the LPS- and LPS+MK-801-treated kidneys at 48 h (n = 3). The lower bar graphs show the ratio of densitometric units (DU) of NR1 to actin (n = 8). Note that MK-801 treatment did not affect renal NR1 expression after LPS treatment.
(JPG)

S1 Table The primer sequences used for real-time RT-PCR. (PDF)

S2 Table Basic body data for groups. Eight rats in each group. BW, body weight; LKW, left kidney weight; Hct, hematocrit.
(PDF)
