# Supplementary figures and images for: Blockade of the N-Methyl-D-Aspartate Glutamate Receptor Ameliorates Lipopolysaccharide-Induced Renal Insufficiency
Source: PLoS One. 2015 Jul 2;10(7):e0132204. doi: 10.1371/journal.pone.0132204 (PMC4489897; doi:10.1371/journal.pone.0132204)

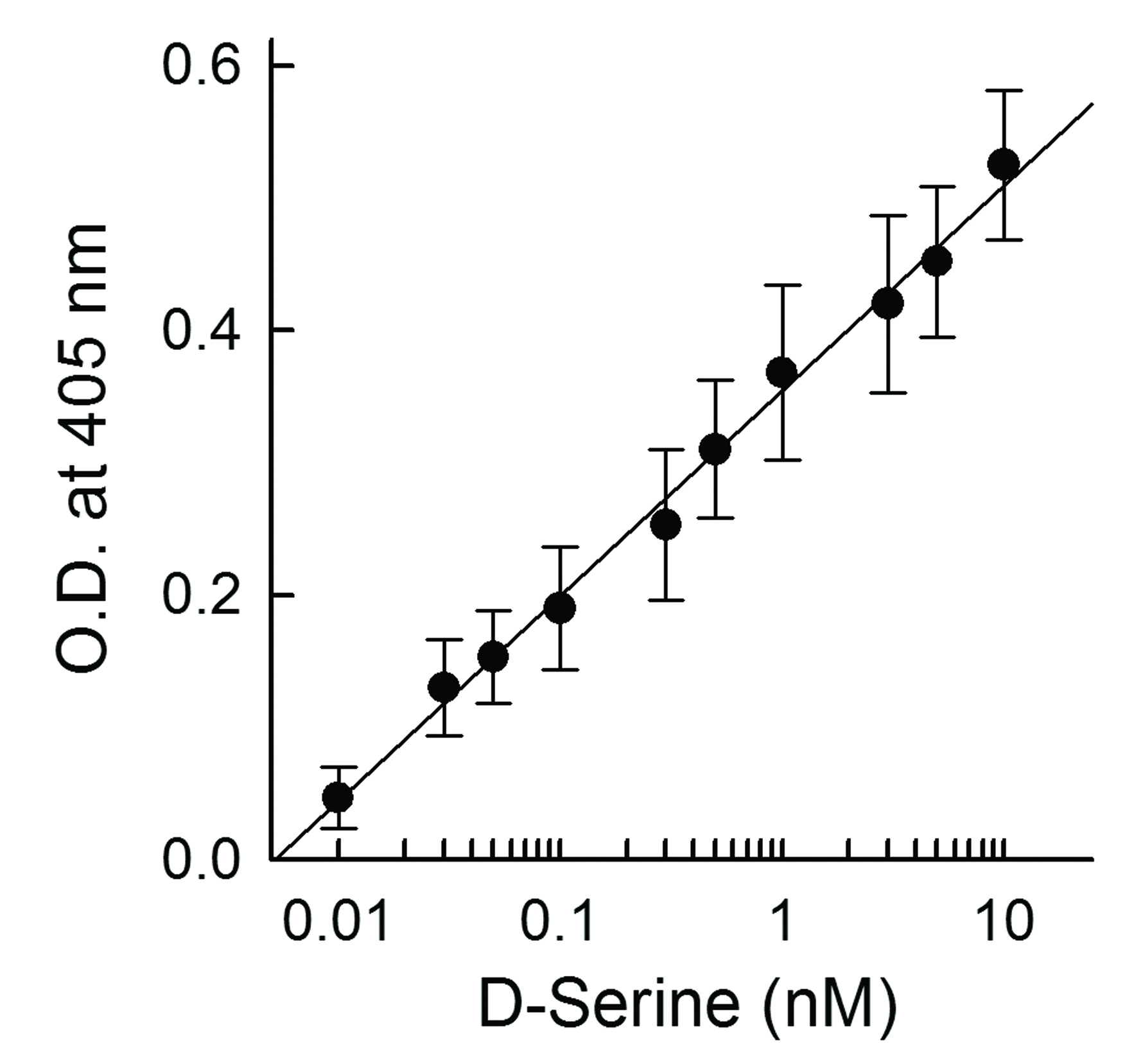

Supplement: S1 Fig — The curve demonstrated a linear relationship between D-serine concentration (within a range of 0.01–10 nM) and optical density (O.D.) at 405 nm. Each data point is representative of five experiments. (JPG) [file pone.0132204.s001.jpg]

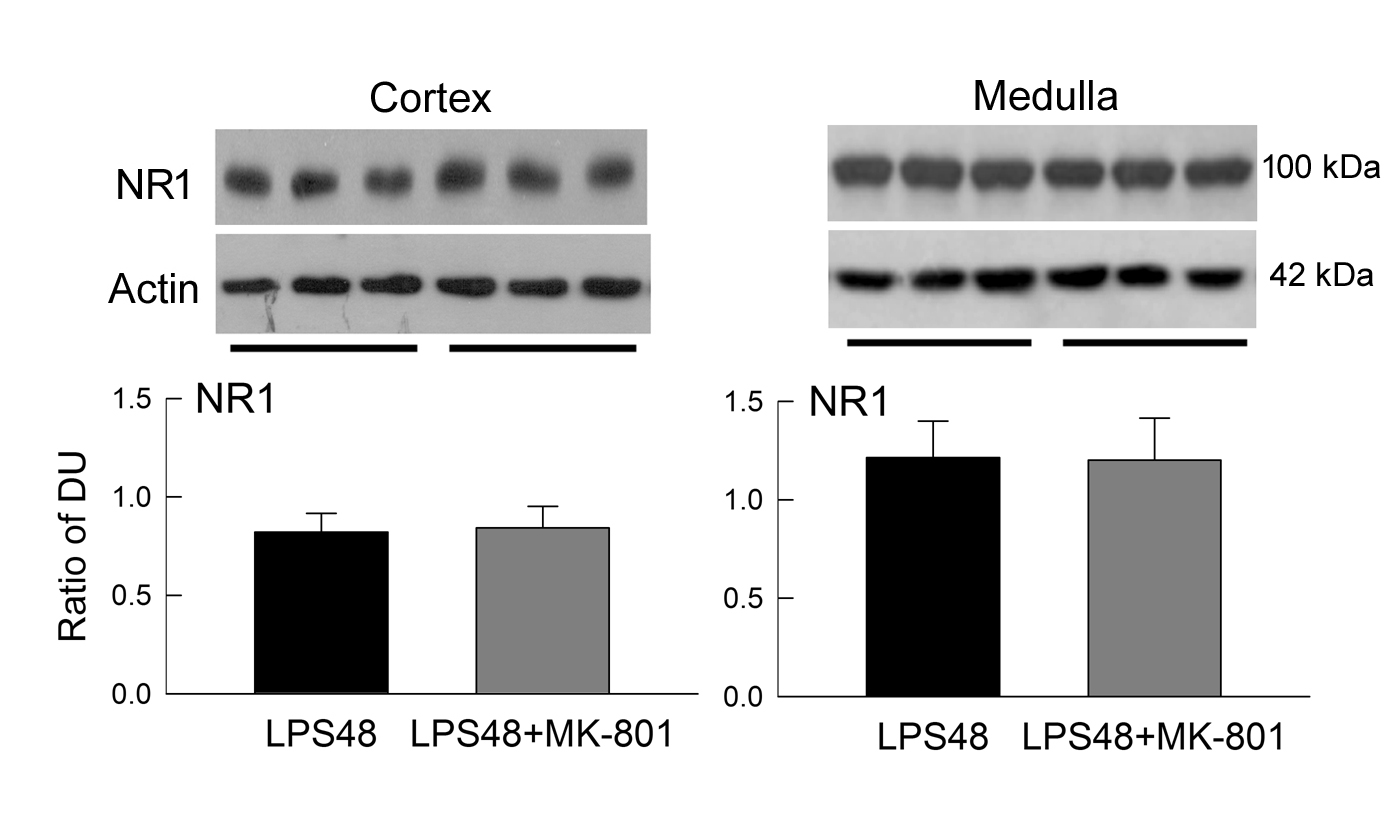

Supplement: S2 Fig — The upper panels show representative blots from the LPS- and LPS+MK-801-treated kidneys at 48 h (n = 3). The lower bar graphs show the ratio of densitometric units (DU) of NR1 to actin (n = 8). Note that MK-801 treatment did not affect renal NR1 expression after LPS treatment. (JPG) [file pone.0132204.s002.jpg]
