# Supplementary material for: Blockade of the N-Methyl-D-Aspartate Glutamate Receptor Ameliorates Lipopolysaccharide-Induced Renal Insufficiency
Source: PLoS One. 2015 Jul 2;10(7):e0132204. doi: 10.1371/journal.pone.0132204 (PMC4489897; doi:10.1371/journal.pone.0132204)
Supplement: S1 Table — (PDF) [file pone.0132204.s003.pdf]

S1 Table. The primer sequences used for real-time RT-PCR.

Species (GenBank entries)	Gene	Primer sequences
Rattus norvegicus (NM_001287423)	NR1	Forward 5’-GAA TGA TGG GCG AGC TAC TCA-3’<br>Reverse 5’-ACG CTC ATT GTT GAT GGT CAG T-3’
Rattus norvegicus (NM_0178008)	GAPDH	Forward 5’-CCC CCA ATG TAT CCG TTG TG-3’<br>Reverse 5’-TAG CCC AGG ATG CCC TTT AGT-3’
Sus scrofa (XM_005659036)	NR1	Forward 5’-ATT CCC CAC CCC CAG CAG AG-3’<br>Reverse 5’-GCC ACA ACC TGG ATC TGG GT-3’
Sus scrofa (NM_001206359)	GAPDH	Forward 5’-ACA CTC ACT CTT CCA CTT TTG-3’<br>Reverse 5’-CAA ATT CAT TGT CGT ACC AG-3’
Canis lups (NM_001008717)	NR1	Forward 5’-GTC CAT CTA CTC AGA CAA GAG-3’<br>Reverse 5’-GAA CCA GAC GCT CGA CTG GT-3’
Canis lups (NM_001003142)	GAPDH	Forward 5’-AAC ATC ATC CCT GCT TCC AC-3’<br>Reverse 5’-GGC AGG TCA GAT CCA CAA CT-3’
