# Supplementary material for: Blockade of the N-Methyl-D-Aspartate Glutamate Receptor Ameliorates Lipopolysaccharide-Induced Renal Insufficiency
Source: PLoS One. 2015 Jul 2;10(7):e0132204. doi: 10.1371/journal.pone.0132204 (PMC4489897; doi:10.1371/journal.pone.0132204)
Supplement: S2 Table — Eight rats in each group. BW, body weight; LKW, left kidney weight; Hct, hematocrit. (PDF) [file pone.0132204.s004.pdf]

S2 Table. Basic body data for groups.

Group	BW (g)	LKW (g)	Plasma $[Na^+]$ (mEq)	Hct (%)
Control	218 ± 8	1.02 ± 0.08	139.2 ± 0.5	43.2 ± 1.1
LPS8	221 ± 7	1.05 ± 0.09	140.6 ± 0.3	42.1 ± 1.3
LPS24	213 ± 9	1.07 ± 0.10	140.9 ± 0.6	44.6 ± 2.1
LPS48	216 ± 10	1.11±0.11	141.1 ± 0.8	43.9±0.9
MK-801	220 ± 9	1.04 ± 0.09	140.5 ± 0.3	41.3 ± 1.6
LPS8+MK-801	225 ± 8	1.06 ± 0.10	140.2 ± 0.4	42.3 ± 1.8
LPS24+MK-801	221 ± 10	0.99 ± 0.08	139.4 ± 0.6	40.6 ± 1.5
LPS48+MK-801	218 ± 7	1.01 ± 0.07	139.1 ± 0.4	41.2 ± 0.9

Eight rats in each group. BW, body weight; LKW, left kidney weight; Hct, hematocrit.
